# Does engagement matter? The impact of patient and community engagement on implementation of cardiovascular health materials in primary care settings

**DOI:** 10.1186/s12875-024-02365-w

**Published:** 2024-04-25

**Authors:** Linda Zittleman, John M. Westfall, Danelle Callen, Alisha M. Herrick, Carolina Nkouaga, Matthew Simpson, L. Miriam Dickinson, Douglas Fernald, Arthur Kaufman, Aimee F. English, W. Perry Dickinson, Donald E. Nease

**Affiliations:** 1 Department of Family Medicine, University of Colorado School of Medicine, Aurora, CO USA; 2 Department of Family and Community Medicine, University of New Mexico School of Medicine, Albuquerque, NM USA; 3 The Center for Health Innovation, New Mexico’s Public Health Institute, Albuquerque, NM USA

**Keywords:** Cardiovascular health, Primary care, Community based participatory research

## Abstract

**Background:**

Engaging patients and community members in healthcare implementation, research and evaluation has become more popular over the past two decades. Despite the growing interest in patient engagement, there is scant evidence of its impact and importance. Boot Camp Translation (BCT) is one evidence-based method of engaging communities in research. The purpose of this report is to describe the uptake by primary care practices of cardiovascular disease prevention materials produced through four different local community engagement efforts using BCT.

**Methods:**

EvidenceNOW Southwest (ENSW) was a randomized trial to increase cardiovascular disease (CVD) prevention in primary care practices. Because of its study design, Four BCTs were conducted, and the materials created were made available to participating practices in the “enhanced” study arm. As a result, ENSW offered one of the first opportunities to explore the impact of the BCT method by describing the uptake by primary care practices of health messages and materials created locally using the BCT process. Analysis compared uptake of locally translated BCT products vs. all other products among practices based on geography, type of practice, and local BCT.

**Results:**

Within the enhanced arm of the study that included BCT, 69 urban and 13 rural practices participated with 9 being federally qualified community health centers, 14 hospital owned and 59 clinician owned. Sixty-three practices had 5 or fewer clinicians. Two hundred and ten separate orders for materials were placed by 43 of the 82 practices. While practices ordered a wide variety of BCT products, they were more likely to order materials developed by their local BCT.

**Conclusions:**

In this study, patients and community members generated common and unique messages and materials for cardiovascular disease prevention relevant to their regional and community culture. Primary care practices preferred the materials created in their region. The greater uptake of locally created materials over non-local materials supports the use of patient engagement methods such as BCT to increase the implementation and delivery of guideline-based care. Yes, patient and community engagement matters.

**Trial registration and IRB:**

Trial registration was prospectively registered on July 31, 2015 at ClinicalTrials.gov (NCT02515578, protocol identifier 15–0403). The project was approved by the Colorado Multiple Institutional Review Board and the University of New Mexico Human Research Protections Office.

## Contribution to the literature

- Community members and patients were able to translate complex medical jargon into locally relevant, actionable messages and materials that supported practice level interventions to improve cardiovascular disease risk reduction.
- Practices were more likely to select messages and materials that were created by community members with similar demographic backgrounds including language, race, and rural/urban geography.
- One-size-fits-all or national efforts may benefit from local translation and modification that maintains scientific integrity but incorporates local culture, values, and language.
- Using a participatory research approach, community members and patients may provide an important addition to practice level implementation efforts.

## Background

Multiple barriers delay the implementation of clinical guidelines and health recommendations as routine practice for patients, community members, and clinicians. Our nation suffers the consequences of missed opportunities for preventive care and underutilized treatments leading to poor health outcomes [1, 2]. Contributing to this delay is the ineffective dissemination of guidelines that do not reach the people that need them most. Medical jargon and difficult concepts hinder uptake by patients and communities [3]. Engaging patients and community members in health research is an important strategy to influence primary care practice implementation of evidence-based guidelines and improve community health outcomes [4, 5]. 

As the number of patients and community members engaged as research partners has increased [6–8], the demand for effective and feasible methods of engaging communities in research has also increased. Evidence-based engagement activities that might inform research are limited. Improving the science of engagement is critical as more researchers seek to understand the impact of patient and community engagement on research and outcomes [9]. 

Boot Camp Translation (BCT) is one evidence-based method of engaging communities in research. BCT combines patient and community expertise with academic researchers to translate evidence-based medical information and clinical guidelines into concepts, messages, and materials that are locally relevant and actionable [10]. BCT deepens the understanding of a health condition among patients and community members so that they are more prepared and motivated to take action. The process informs health care professionals about patient and community members’ perspective on a health issue. For over a decade, BCT has been used in projects addressing a wide variety of health topics including diabetes, asthma, mental health [11–15]. BCT is built on the principles of community-based participatory research (CBPR) and uses this community engagement approach to develop, implement, and test interventions and disseminate results [16–18]. 

One of the foundations of BCT is that a one-size-fits-all approach to health messages and dissemination strategies is not effective in reaching desired populations and influencing health behaviors. BCT strives to incorporate local culture and assets while prioritizing community preferences. The results are scientifically valid, evidence-based messages and materials that are relevant to and preferred by communities. This paper describes the uptake of messages and materials created using the patient engaged BCT process by primary care practices in the multi-state EvidenceNOW Southwest study [19, 20]. We sought to answer the question: did primary care practices favor messages and materials created by their local BCT, or did they select a wider variety of messages and materials created by any of the four BCTs conducted in the EvidenceNOW Southwest study?

## Methods

EvidenceNOW Southwest (ENSW) was a trial to test the impact on cardiovascular disease (CVD) of primary care practice transformation focused on patient, family, and community engagement. Practice transformation is the broad term for supporting practices making changes to improve quality, increase patient satisfaction, and reduce costs. Practice transformation includes programs like the medical home, practice facilitation and coaching, and population health. ENSW was a collaborative effort between the University of Colorado and the University of New Mexico that enrolled small and medium-sized primary care practices (with 1–15 clinicians). ENSW used geographic-based covariate constrained randomization to allocate 26 distinct geographic regions in Colorado and 16 in New Mexico to the standard or enhanced study arm. Over 200 practices were included in the study based on their location within the geographically distinct regions. Trial registration for the overall ENSW study was prospectively registered on July 31, 2015 at ClinicalTrials.gov (NCT02515578, protocol identifier 15–0403). A total of four regional BCTs were conducted in enhanced study arm regions to translate the complex medical jargon in CVD prevention guidelines into locally relevant actionable messages, materials, and implementation strategies for CVD prevention. Two BCT’s were held in each state with emphasis placed on engaging community stakeholders from urban and rural backgrounds. Unlike the standard arm, practices in the enhanced study arm received support for patient and community engagement and were also given the opportunity to receive any of the materials created by the four BCT groups. Because of its study design, ENSW offered one of the first opportunities to explore the impact of the BCT method by describing the uptake by primary care practices of health messages and materials created locally vs. study-wide.

Results of the overall EvidenceNOW SW randomized trial, which are not the focus of this report, showed an increase in evidence-based testing and treatment for cardiovascular disease and are reported elsewhere [20]. 

Participating practices were randomized to a standard or an enhanced study arm. The control or standard arm intervention package provided to all practices included evidence-based CVD prevention guidelines, with a focus on aspirin use, blood pressure control, cholesterol management, and smoking cessation (as selected by the funding agency) along with support from a trained practice facilitator/coach and attendance at statewide Collaborative Learning Sessions. Practices in the enhanced study arm also received training and support for patient and community engagement in their practice transformation efforts. A second component of the patient and community engagement was the use of Boot Camp Translation to translate the complex medical jargon in CVD prevention guidelines into locally relevant actionable messages and materials.

Each BCT engaged 8–10 patients andcommunity members, 1–2 public health professionals, and 1–2 providers orpractice staff. Four BCTs were conducted: 1) rural Hobbs in southwest New Mexico; 2) urban Albuquerque, New Mexico; 3) rural northeast Colorado; and 4) urban Denver, Colorado. The BCT processes lasted six to eight months each, and each generated two to four unique products. Common themes emerged across the four BCT groups, however each BCT also generated unique messages and materials that reflected local culture, priorities, and communication strategies. A full description of the BCT process and resulting materials is reported elsewhere [21]. 

As part of the larger clinical trial, this BCT element was community-based participatory research and participants in Boot Camp Translation were study partners, not research subjects. Boot Camp Translation participants served as research advisors and all research was performed in accordance with the principles of the Belmont Report and the United States Federal Policy for the Protection of Human Subjects, HHS regulations, 45 CFR part 46. The project was approved by the Colorado Multiple Institutional Review Board and the University of New Mexico Human Research Protections Office.

### Distribution of BCT materials

Each practice in the enhanced arm was associated with one of the 4 BCTs based on their geographic location. Only enhanced study arm practices had access to materials created through the BCT process, therefore only enhanced study arm practices are included in this analysis. Other than the standard guidelines that were distributed to all practices, no other materials were provided to practices other than the BCT materials. Any of the BCT materials, regardless of where they were developed, were available at no cost to these practices. Practices learned about all the BCT materials through Collaborative Learning Sessions which included breakout sessions for enhanced arm practices about the materials. Samples were on display and enhanced practice staff were trained how to select and order materials through an online system. Practice staff, not research study team members or practice facilitators, ordered materials for their own practice. The online descriptions of the materials did not identify the BCT that developed the materials. Orders were tracked using the online system. A team at each academic institution processed the orders and shipped or delivered materials during in-person visits to practices in a timely manner. Because practices had access to any of the materials, we were curious whether practices would select materials from their local BCT or from other BCTs.

### Data collection and analysis

The main outcome of this analysis is the *uptake of materials* defined by the proportion of practices that ordered any BCT product, the number of orders per practice, and preferences for materials. Preference is defined two ways: (1) the ordering of BCT materials based on the practice characteristics and (2) the ordering of BCT materials based on the practice’s affiliated BCT. We report differences in uptake by practice characteristics that could influence these outcomes: location (urban vs. rural), ownership (Federally Qualified Health Center or FQHC, clinician-owned, and hospital-owned), and size (≤ 5 clinicians and > 6 clinicians). Data on uptake came from the online product ordering system at the ENSW website and order logs.

Descriptive statistics (means, standard deviation, frequencies) were generated for practice and BCT types and characteristics. The primary outcome variable for overall analysis was total *number of BCT orders* for each practice in the ENSW enhanced study arm. The outcome variables for preference associations were total number of orders for materials developed by the rural BCTs combined, the urban BCTs combined, and the number of orders from practices associated with each of the four BCTs. Independent variables include *practice* characteristics described above (location, ownership, size) and the practices’ affiliated BCT. Associations between product orders and practice characteristics were examined using adjusted Poisson regression analyses. All analyses were performed using SAS software (Version 9.4, SAS Institute Inc., Cary, NC, USA).

## Results

### Practice characteristics and uptake of materials

The EvidenceNOW Southwest enhanced study arm consisted of 82 practices. These practices included a mix of ownership types, sizes, and locations. As shown in Tables 1 and 84% of the practices were located in urban areas, 59% were clinician-owned, and 63% had five or fewer clinicians. Of the 82 practices, 43 (or 52%) placed at least one order for CVD prevention materials created using the BCT process. Many practices ordered a variety of materials, and all materials were ordered at least once. A similar proportion of practices in each state ordered materials created by a BCT (35/68 = 51% in Colorado, 8/14 = 57% in New Mexico).

Table 2 shows the number of orders for each product overall and by practice location. Results in Table 3 indicate a trend towards more total orders (uptake) from rural practices than urban practices (mean number of orders rural = 3.6 vs. urban 2.6, *p* = .05). Clinician owned practices placed more orders than FQHCs and hospital-owned practices (mean number of orders = 3.2, 1.6, and 1.8, respectively, *p* = .0003). There was no association between practice size and the number of orders.

### Preference for BCT materials based on practice characteristics

Rural practices were more likely to order rural BCT products than urban practices (mean number of orders = 2.6 vs. 1.5, *P* = .004). Further, clinician owned practices ordered more materials. There was no association between practice size and preference for urban or rural products.

### Orders for BCT materials based on the practice’s affiliated BCT

We were particularly curious whether practices preferred materials developed from their local BCT. While a wide variety of materials were ordered by many of the enhanced arm practices, there was a pattern of preference for materials developed by their local BCT (Table 4). Practices in rural Colorado placed significantly more orders for BCT materials developed in the rural Colorado BCT (mean number of orders for rural Colorado materials = 3.3 at rural Colorado practices vs. 1.1 at all other practices, *P* < .0001). Rural New Mexico practices and urban New Mexico practices also preferred materials from their affiliated BCT (0.83 vs. 0.29, *P* = .04; and 1.63 vs. 0.58, *P* = .001, respectively). While urban Colorado practices ordered more urban Colorado BCT materials, because these urban Colorado practices took advantage of the opportunity to order any materials, this preference did not reach statistical significance.

## Discussion

This study helps address the call from researchers to measure the impact of community engagement in research [22]. The importance of engaging communities in translational health research and in the development of practice-level interventions that support the implementation of evidence-based guidelines is increasingly reflected in funding agencies’ proposal requirements. Multiple approaches are available to engage patients and community members in research, including BCT. The breadth and depth of the literature detailing these approaches is growing, including descriptions of strategies across the stages of the research, terminology and definitions, the frequency with which strategies are used, and process evaluations [23]. While efforts are emerging to evaluate the impact of community engagement beyond process evaluation [24–26] much more research is needed to provide evidence that supports the various approaches to engage communities.

Overall uptake of BCT materials was very good, with 52% of practices placing at least one order for materials. This study also provides insight into the types of education materials on CVD that practices might select and incorporate into care for their patients. We found that rural practices were more likely to order products created by rural BCTs, and practices preferred products from their affiliated BCT. Other large-scale campaigns, such as Million Hearts® Campaign, while reporting change in CVD events, do not include information on which elements of the intervention were used [27]. Practice utilization of materials and patient-level outcomes is beyond the scope of this manuscript, however, our results address an important question around reach of educational and action-oriented CVD prevention products. Materials created with community members who know the local culture and language was associated with greater uptake in associated practices than materials created elsewhere. Our results support the premise that “local” matters.

Different BCT materials were designed with different messages. Some BCT products, such as the “One Heart” poster, the “Big Sale” poster, and the “Cuidalo” poster, were designed for mass exposure. Fewer quantities were needed at each practice to reach a large number of patients. Other materials were designed to be used by individual patients, such as the Heart Chart, Recipe Cards, One Heart Checklist, and Prevent Second Chances Map. For example, a practice might order 200 Heart Charts, but only 3–4 One Heart posters. For this reason, we used the “order” as the unit of analysis versus the quantities ordered.

Uptake of BCT materials might have been influenced by external factors. In a small number of practices, clinic or health system regulations may have restricted the types of materials that can be used. The organizational review and approval processes may have reduced the amount of time practices had to order materials. Engaged clinic leadership is the first of the 10 building blocks of high-performing primary care [28] and can significantly influence how practices respond to, adopt, and sustain new resources or innovations and any subsequent protocol and work flow changes. In this case, disengaged or disinterested leadership may have limited some practices from ordering BCT materials. The urban Colorado practices ordered a wide range of materials. This may be because urban Colorado has greater heterogeneity of communities and practices, leading to more interest in some of the other BCT materials with more cultural connection.

A limitation of this study is the small number of BCTs conducted for a fairly large number of practices across the participating rural and urban regions. Ideally, more BCTs would be conducted in smaller regions and among unique cultural groups. However, we needed to balance the granularity of the BCT partnerships with the project’s capacity to pay for and conduct BCTs. We did not collect data on how BCT materials were used in the practices. However, it is reasonable to believe that practices distributed and used the materials they ordered. A strength of this study is that practice staff selected and ordered the materials they would implement in their practice from among all materials produced by the four BCTs. While each BCT may have included a participant from a local practice, there were over 80 practices in the enhanced arm. Therefore, their participation is unlikely to have influenced the results. The practices themselves were not involved in the BCT process and had access to all materials, providing the opportunity to measure their preferences. Our analysis focused on the enhanced study arm and because of the geographic randomization, there was little chance for cross contamination between practices. The geographic areas for the BCTs were selected to avoid the chance of cross contamination during the BCT process (e.g., urban Albuquerque versus Hobbs in far southwest New Mexico).

## Conclusions

Yes, patient and community engagement matters. Community members and patients can provide an important addition to the creation of interventions and programs designed to support and improve practice-level prevention programs. In this study, patients and community members in four different regions successfully used Boot Camp Translation to generate messages and materials around cardiovascular disease prevention relevant to their regional community culture. Primary care practices preferred the materials created in their region. The greater uptake of locally created materials over non-local materials supports the use of methods such as BCT to engage patients and community members in health research and to facilitate the implementation and delivery of guideline-based care.

**Table 1:** Characteristics of practices in the enhanced study arm (*N* = 82)

Practice characteristic	Description	Practices n (%)
Location	Urban	69 (84)
	Rural	13 (16)
Ownership	FQHC*	9
	Hospital owned	14
	Clinician owned	59
Size	≤ 5 clinicians	63
	6 + clinicians	19
State	Colorado	68
	New Mexico	14

*Federally Qualified Health Center.

**Table 2:** Orders for CV disease prevention products created using Boot Camp Translation

Product name	BCT of origin	Total orders	Orders from the 7 Rural CO practices	Orders from the 61 Urban CO practices	Orders from the 6 Rural NM practices	Orders from the 8 Urban NM practices
Heart chart	Rural CO	31	8	21	0	2
Recipe cards	Rural CO	78	15	51	5	7
One heart brochure	Urban CO	10	1	9	0	0
One heart checklist	Urban CO	15	2	13	0	0
One heart poster	Urban CO	9	1	8	0	0
Big sale poster	Rural NM	13	1	10	1	1
Our bodies magnet	Rural NM	2	0	2	1	2
NM Fan	Rural NM	9	0	1	3	5
Cuidalo grocery bag	Urban NM	3	0	3	0	4
Cuidalo poster	Urban NM	8	1	3	1	3
CVD dashboard	Urban NM	16	2	14	0	6
Prevent second chances map	Urban NM	16	2	14	3	0
Total # of orders		210	33	149	14	30

BCT = Boot Camp Translation. CVD = Cardiovascular disease. CO = Colorado. NM = New Mexico.

**Table 3:** Association between practice characteristics and uptake of any Boot Camp Translation materials

Practice characteristic	Description	Mean # of orders (SD)	P value
Location	Rural (*n* = 13)	**3.6 (4.0)**	**0.053**
	Urban (*n* = 69)	2.6 (3.7)	
Ownership	FQHC (*n* = 9)	1.6 (2.1)	**0.0003**
	Hospital owned (*n* = 14)	1.8 (3.0)	
	Clinician owned (*n* = 59)	**3.2 (4.0)**	
Size	≤ 5 clinicians (*n* = 63)	2.6 (3.5)	0.273
	6 + clinicians (*n* = 19)	3.2 (4.3)	

**Table 4:** Practices’ preference for BCT products created by practices’ affiliated BCT

BCT	Practice location	Product order type	# of Orders Mean (sd)	P value
Rural CO	Rural CO (*n* = 7)	Rural CO BCT	3.3 (2.6)	**< 0.0001**
	All other practices (*n* = 75)		1.1 (2.3)	
Urban CO	Urban CO (*n* = 61)	Urban CO BCT	0.49 (0.85)	0.069
	All other practices (*n* = 21)		0.19 (0.60)	
Rural NM	Rural NM (*n* = 6)	Rural NM BCT	0.83 (0.98)	**0.035**
	All other practices (*n* = 76)		0.29 (0.63)	
Urban NM	Urban NM (*n* = 8)	Urban NM BCT	1.63 (1.60)	**0.001**
	All other practices (*n* = 74)		0.58 (1.21)	

BCT = Boot Camp Translation. CO = Colorado. NM = New Mexico.

## Data Availability

The datasets analyzed during the current study are available from the corresponding author on reasonable request.
